# Acute effects of Expiratory Positive Airway Pressure (EPAP) on different levels in ventilation and electrical activity of sternocleidomastoid and parasternal muscles in Chronic Obstructive Pulmonary Disease (COPD) patients: a randomized controlled trial

**DOI:** 10.1590/bjpt-rbf.2014.0190

**Published:** 2016-09-22

**Authors:** Dannuey M. Cardoso, Guilherme A. F. Fregonezi, Renan T. Jost, Ricardo Gass, Cristine L. Alberton, Isabella M. Albuquerque, Dulciane N. Paiva, Sérgio S. M. Barreto

**Affiliations:** 1 Programa de Pós-graduação em Ciências Médicas, Universidade Federal do Rio Grande do Sul (UFRGS), Porto Alegre, RS, Brazil; 2 Departamento de Educação Física e Saúde, Universidade de Santa Cruz do Sul (UNISC), Santa Cruz do Sul, RS, Brazil; 3 Programa de Pós-graduação em Ciências Pneumológicas, UFRGS, Porto Alegre, RS, Brazil; 4 Laboratório de Desempenho PneumoCardioVascular & Músculos Respiratórios, Universidade Federal do Rio Grande do Norte (UFRN), Natal, RN, Brazil; 5 Laboratório PneumoCardioVascular, Hospital Universitário Onofre Lopes, Empresa Brasileira de Serviços (EBSERH), Natal, RN, Brazil; 6 Escola de Educação Física, Universidade Federal de Pelotas (UFPel), Pelotas, RS, Brazil; 7 Programa de Pós-graduação em Reabilitação Funcional, Departamento de Fisioterapia e Reabilitação, Universidade Federal de Santa Maria (UFSM), Santa Maria, RS, Brazil; 8 Programa de Pós-graduação Mestrado em Promoção da Saúde, UNISC, Santa Cruz do Sul, RS, Brazil; 9 Serviço de Pneumologia, Hospital de Clínicas de Porto Alegre (HCPA), Porto Alegre, RS, Brazil

**Keywords:** chronic obstructive pulmonary disease, electromyography, intercostal muscles, positive-pressure respiration, respiratory muscles, physical therapy

## Abstract

**Objective:**

To investigate the acute effects of EPAP on the activity of sternocleidomastoid (SCM), parasternal muscles and ventilatory parameters in COPD patients.

**Method:**

Twenty-four patients with COPD were studied using surface electromyography (*sEMG*) and a ventilometer. Patients were randomly assigned to EPAP 10 cmH_2_O-EPAP_10_ or 15 cmH_2_O-EPAP_15_ for 20 minutes.

**Results:**

The parasternal muscle *sEMG* activity increased during EPAP_10_ and EPAP_15_; however, a greater and significant increase was observed with EPAP_10_ (mean between-group difference: 12.5% RMS, 95% CI: 9.5 to 15.4, *p*<0.001). In relation to the baseline, at 10 and 20 minutes and upon recovery, respectively parasternal activity increased by 23.9%, 28.9% and 19.1% during EPAP_10_ and by 10.7% at 10 and 20 minutes and upon recovery, respectively, 11.4% and 6.9% during EPAP_15_ at 10 and 20 minutes and upon recovery, respectively. The *sEMG* activity of SCM muscle showed an opposite pattern, increasing with EPAP_15_ and decreasing with EPAP_10_ (mean between-group difference: 15.5% RMS, 95% CI: 12.6 to 18.4, *p*<0.001). SCM muscle activity during EPAP_15_, increased by 4.8% and 6.1% at 10 and 20 minutes and decreased by -4.0% upon recovery compared to decreases of –5.6%, –20.6% and –21.3% during EPAP_10_ at 10, 20 minutes, and recovery. Ventilation at both EPAP intensities promoted significant reductions in respiratory rate (RR) and dyspnea, more pronounced in EPAP_15_: RR (mean between-group difference: –3,8bpm, 95%CI: –7,5 to –0,2, *p*=0,015) and dyspnea (mean between-group difference: –1.01, 95%CI: –1.4 to –0.53, *p*=0.028) .

**Conclusion:**

In COPD patients, the use of EPAP_10_ was more effective in reducing accessory inspiratory activity and increasing parasternal activity, which was accompanied by an improvement in ventilation and a reduction in dyspnea.

## BULLET POINTS

• In COPD patients, respiratory muscles showed different patterns depending on expiratory load;
• The parasternal muscle was more active during breathing against EPAP_10_;
• During EPAP_15_, COPD patients developed improper respiratory muscle recruitment patterns;
• EPAP 10 results in a better physiologic breathing pattern compared to EPAP_15_.

## Introduction

Chronic obstructive pulmonary disease (COPD) is characterized by chronic obstruction of air flow and reduced aerobic capacity of the peripheral muscles secondary to alterations in ventilatory mechanics^1^. A common feature in a COPD respiratory system is the presence of dynamic hyperinflation that is frequent at rest and increases with exercise^2^.

Another mechanical change in the respiratory system is the flattening of the diaphragm resulting from an increase in physiological dead space and dynamic hyperinflation that contributes to respiratory mechanical inefficiency. This change results in greater energy expenditure required by the respiratory muscles, and is also related to the presence of dynamic intrinsic positive end-expiratory pressure (i.e., dynamic intrinsic positive end-expiratory pressure [PEEPi])^3^ ^,^ ^4^.

The dynamic PEEPi is a positive end-expiratory alveolar pressure that is not extrinsically applied and occurs at the beginning of inspiration in a volume above relaxation volume when inspiratory muscles markedly reduce the pleural pressure. In this case, the mechanical breathing that is necessary to generate the ventilation demands more work. In COPD patients, the metabolic cost of breathing is assigned to the expiratory muscles that contract during exhalation^5^. This phenomenon reduces the capacity of expiratory muscles to increase ventilation because of an expiratory flow limitation which promotes an imbalance in respiratory muscle work^5^ ^,^ ^6^.

In the past, several devices have been used by respiratory physical therapists to improve ventilation and to preserve physiological levels of lung volume. Expiratory pressure delivery systems employing valve devices (e.g. positive expiratory pressure [PEP]) or a face mask (e.g. expiratory positive airway pressure [EPAP] devices) have been used in COPD patients, particularly to assist in elimination of respiratory secretions^7^ ^,^ ^8^. Additionally, an expiratory pressure device increases the resistance during the expiratory phase and induces a reduction in minute ventilation (V_E_), respiratory rate (RR) and physiological dead space. Furthermore, it improves the length/tension ratio of respiratory muscles, making them more efficient^9^.

The purpose of this study was to assess the acute effects of EPAP at two different intensities, 10 cmH_2_O (EPAP_10_) and 15 cmH_2_O (EPAP_15_), on the electrical activity levels of the sternocleidomastoid (SCM) and parasternal intercostal muscles and on ventilation in patients with stable COPD. The hypotheses tested was the two different loads of EPAP would improve the coordination of the respiratory muscles studied (i.e.parasternal and SCM) and would enhance ventilation.

## Method

### Study design

A double blind randomized controlled trial was conducted in the Pulmonology Service Unit at the Porto Alegre University Hospital from November 2009 to September 2011. All patients were recruited from the COPD ambulatory unit of the same hospital. At a clinical visit to a secondary clinical setting, subjects went through an interview, an anthropometric evaluation, a spirometry assessment, a respiratory muscle function test and an EPAP at different intensities along with surface electromyography (sEMG) testing of the sternocleidomastoid (SCM) and the third right intercostal muscle.

### Patients

Eligibility criteria for the study included patients with COPD (GOLD stages II-III)^1^ who accepted the study proposal and exhibited clinical stability of the disease with no signs of exacerbation during the 30 days prior to enrollment. The exclusion criteria were the current use of supplemental oxygen, hemodynamic instability, or a body mass index (BMI) >30 Kg/m^2^. All subjects signed an informed written consent, which was approved by the Ethics Committee of Hospital de Clínicas de Porto Alegre, Universidade Federal do Rio Grande do Sul (UFRGS), Porto Alegre, RS, Brazil (protocol number 09-500). This trial was registered at www.ClinicalTrials.gov (Identifier: NCT01111487) in November 2009.

### Randomization and blinding

A computer-generated list of random numbers was used, and a randomization sequence was created by the software Random Number Generator (Pro v2.00, Segobit, Issaquah, WA, USA). Each subject was assigned to one of two groups (EPAP_10_ or EPAP_15_) through a sequence that was stratified accordance with the severity of disease, with a ratio of allocation of 1: 1 using random block sizes of 2 and 4. All participants received the intervention by two physical therapists blinded, which did not participate in the evaluation process. The assessments were also blinded and performed by only one physical therapist who was not involved in the randomization process. Randomization was performed by an external collaborator, without direct involvement in the study. After randomization, the level of pressure selected was covered in the post end-expiratory pressure (PEEP) valve was covered with duct tape by the physical therapist responsible for the randomization. Thus the patient and the physiotherapist who applied the mask EPAP were not aware of the pressure level used.

### Intervention

The anthropometric data, pulmonary function and respiratory muscle strength of the patients were evaluated. Weight and height were assessed by a digital scale with a stadiometer (2096PP, Toledo, São Bernardo do Campo, SP, Brazil), and body mass index (BMI) was successively calculated on the first day. After screening for exclusion and inclusion criteria, the patients who gave informed consent were included in group that used the EPAP of 10 cmH_2_O (EPAP_10_) or 15 cmH_2_O (EPAP_15_). The following week, the patients returned for sEMG measurements that were recorded with the patient in a sitting position, where an initial measurement was taken during spontaneous breathing (i.e. baseline or control situation), followed by a maximal voluntary isometric contraction (i.e., an inspiration maximum for signal normalization). After the EMG signal was reestablished to near-basal levels, 10 or 15 cmH_2_O EPAP was applied for 20 minutes via a face mask (Vital Signs^®^, New Jersey, USA) containing a unidirectional valve with a PEEP-generating expiratory resistance mechanism (Vital Signs^®^, New Jersey, USA). The sEMG signal was captured at 10 and 20 minutes of application and 10 minutes after the removal of the mask to determine whether there was a sustained effect on the muscles.

### Measurements

In this study sEMG and ventilation (tidal volume) were considered to be the primary outcomes. All the other’s variables were considered to be secondary outcomes.

### Surface EMG recordings

The sEMG was conducted using circular surface electrodes with a radius of 15 mm, using a bipolar configured Meditrace 100 pediatric electrode (Tyco Healthcare, QC, Canada). The signal was pre-amplified and connected to a differential surface sensor (model SDS500) with a clamp connection using a 100-fold gain, filter frequencies ranging from 0.1 to 500 Hz or 1000 Hz and a 2-pole Butterworth architecture. A 2-cm space was maintained between electrodes to reduce crosstalk^10^. The signal was captured by a surface electromyography device Miotool 400 (MIOTEC, RS, Brazil) composed of a 2-channel system with 14 bits of resolution, a sampling frequency of 2000 Hz per channel, a common mode rejection of 110 decibels (db), a low noise level below 2 Least Significant Bit (LSB), and a 100-fold gain amplifier.

To remove dead skin cells and enhance the EMG signal, cotton swabs moistened with alcohol were used to disinfect and roughen the skin^11^. The electrodes containing a conductive gel were affixed with adhesive tape to the middle line of the muscle, with their detection surface perpendicular to the muscle fiber^12^. The location of the muscle of interest was based on the palpation of the central portion for the right SCM, 3 cm above its anterior head in the posterior triangle of the neck, during segmental neck flexion against manual resistance. The participant was asked to perform a brief isometric flexion contraction (3-5s) of the neck to confirm that the electrodes were in the correct position^13^. Similarly, an electrode was placed in the third right intercostal space, near the sterna border, for the right parasternal muscle^14^.

The curve corresponding to the EMG signal of the inspiratory phase was obtained by observing the intra-mask pressure (MVD300, RS, Brazil). Specifically, the inspiratory phase was defined as the period beginning 1 second after the absence of intra-mask expiratory pressure was detected and ending 1 second before the presence of any pressure level was detected within the mask^15^. The root mean square (RMS) value of the EMG signal consisting of 2 minutes in the middle of a 4-minute window was used for analysis.

The captured signal by the Miograph software (MIOTEC^®^, RS, Brazil) was then exported for analysis (SAD 32) by a fourth-order Butterworth high-pass filter with a cutoff frequency of 20 Hz, a third-order Butterworth low-pass filter with a cutoff frequency of 1000 Hz, and a 50 to 60 Hz band-pass filter^15^ ^,^ ^16^. The EMG activity values during the maximal inspiratory pressure (MIP) measurement were used to normalize the SCM and the parasternal muscle activity, and the values of the EMG were described as the maximum activation percentage of the respective muscle (%RMS).

### Pulmonary function

Subjects were instructed about the procedures to be performed during the spirometry assessment. The spirometer was routinely calibrated every day using a 3-L syringe, according to ambient temperature conditions, as well as standardizations for measures, were in accordance with the Brazilian Society of Tisiology and Pneumology^17^. A minimum of 3 and a maximum of 8 tests were conducted with a 1-min interval between each test to get 3 reproduceable curves. A spirometer (Jaeger-v4.31, Wuerzburg, Germany) was used to measure forced expiratory volume in 1 second (FEV_1_) and forced vital capacity (FVC). Three reproducible tests were performed, and the best curve was considered for the study. Results were expressed both as absolute and as percent-of-predicted values^18^.

### Respiratory muscle strength

The respiratory muscle strength was measured using a MVD 300 digital manometer (MDI^®^, RS, Brazil). The tests were conducted with patients in the sitting position and the patients made an effort to blow out against the occluded valve^19^_._ Before each test, the patients were thoroughly instructed regarding the procedures, and the results obtained were assessed in their absolute values. The figure considered for data analysis was the highest value among the three tests if it did not differ more than 10% from the second highest value in descending order. Maximum expiratory pressure (MIP) was obtained with the patient breathing in from residual volume (RV) to total lung capacity (TLC), and maximal expiratory pressure (MEP) was recorded from TLC to RV. MIP and MEP were expressed in both absolute and percent-of-predicted values using reference values obtained for the Brazilian population^20^.

### Tidal volume and vital signs

The tidal volume (V_T_) assessment was performed using an analog ventilometer (RM121 Respirometer, Ohmeda, Tokyo, Japan), while respiratory rate (RR) was determined by counting the breaths per minute. The VT and RR data were visually checking on the device and captured at 10 and 20 minutes of the test for 1 minute. According to the equipment’s manufacturers, the device is capable of good precision in current ventilation, with 3-4% variation (continuous flow) depending on the volume LPM (liters per minute): 3% for volumes greater than 5 LPM and 4% by volume 4 LPM. Additionally, it has a low resistance of approximately 2 cmH_2_O^21^ ^-^ ^25^.

The peripheral oxygen saturation (SpO_2_) and heart rate (OxiMax, Puritan Bennett™, MA, USA) were measured, and patients were also asked about the sensation of dyspnea, measured using the modified Borg scale^26^. These variables were evaluated under spontaneous breathing conditions, i.e., before applying EPAP and immediately after its removal.

### Statistical analysis

The statistics analysis was performed using SPSS software, version 20.0. The data were screened for normality using the Shapiro-Wilk test. The physiological variables, V_T_ and the Borg scale were compared using ANCOVA. The effect of applying the EPAP on the SCM and parasternal muscles over time was compared between the groups using a two-way ANOVA, considering the intensities, time, and interaction factors with the *Bonferroni post-hoc* test. The anthropometric, physiological and demographic characteristics and lung function (pre-intervention) were compared between groups using Student’s t-test, except for gender, which was compared using the chi-square test (*p*<0.05). The software G*Power (version 3.1.9.2) was used to calculate the sample size and power of the study. A t-test for independent variables and a significance level of 5% were applied. The data from a pilot study of 4 subjects, with means and standard deviations, were used in each group. Standard deviation of *sEMG* signal from the parasternal muscle after 20 minutes of EPAP application was used. A sample of 6 subjects in each group showed a power of 99% to detect a significant difference in the *sEMG* signal of parasternal muscle after 20 minutes of an EPAP application of 10 between the groups.

## Results

**Figure 1:**
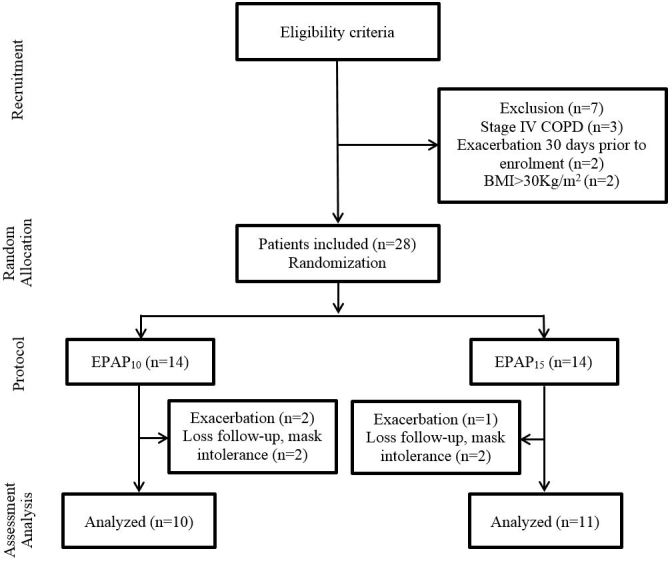
Flowchart of study. COPD: Chronic Obstructive Pulmonary Disease; BMI: Body mass index; EPAP: Expiratory Positive Airway Pressure.

Thirty-five patients were enrolled in the study. Seven patients were not randomized to a treatment group because they did not meet the inclusion criteria during the screening procedures and seven others were lost during the follow-up to application of EPAP (Flowchart, Figure 1). The power achieved with a post-hoc test was calculated with the *sEMG* data from the parasternal muscle after 20 minutes of EPAP, and an effect size of d=6.35 and a power (*1-ß err prob)*= 1.0 were found. Patient demographics are described in Table 1.

**Table 1:** Baseline characteristics by 2 groups of COPD patients, one group receiving EPAP10 & the other EPAP15.

Anthropometric	EPAP_10_	EPAP_15_
Gender (male/female	6/4	3/7
Age (y)	58.2±8.7	57.3±6.7
Weight (Kg)	62.6±13.8	66.8±8
Height (cm)	164.8±9.3	163.3±4.5
BMI (Kg/m^2^)	22.7±3.2	25.0±3.5
Pulmonary Function and Respiratory Muscle Strength		
FVC (%pred.)	62.5±17.8	65.7±16.5
FEV_1_ (%pred.)	42.7±15.0	47.8±16.3
FEV_1_ /FVC (%pred.)	69.4±17.6	67.7±15.4
MIP (cmH_2_O)	70±19.5	62.2±26.2
MIP (%pred)	72.8±16.2	80.5±24.8
MEP (cmH_2_O)	88.5±27.4	68.8±17.5
MEP (%pred)	87.2±22.5	78.4±19.7

BMI: Body mass index; FVC: Forced vital capacity; FEV_1_: Forced expiratory volume in the first second; FVC/FEV_1_: Ratio between forced vital capacity and forced expiratory volume in the first second; MIP: Maximum inspiratory pressure; MEP: Maximum expiratory pressure.

### Effect of EPAP on sEMG of parasternal and sternocleidomastoid muscles

**Figure 2:**
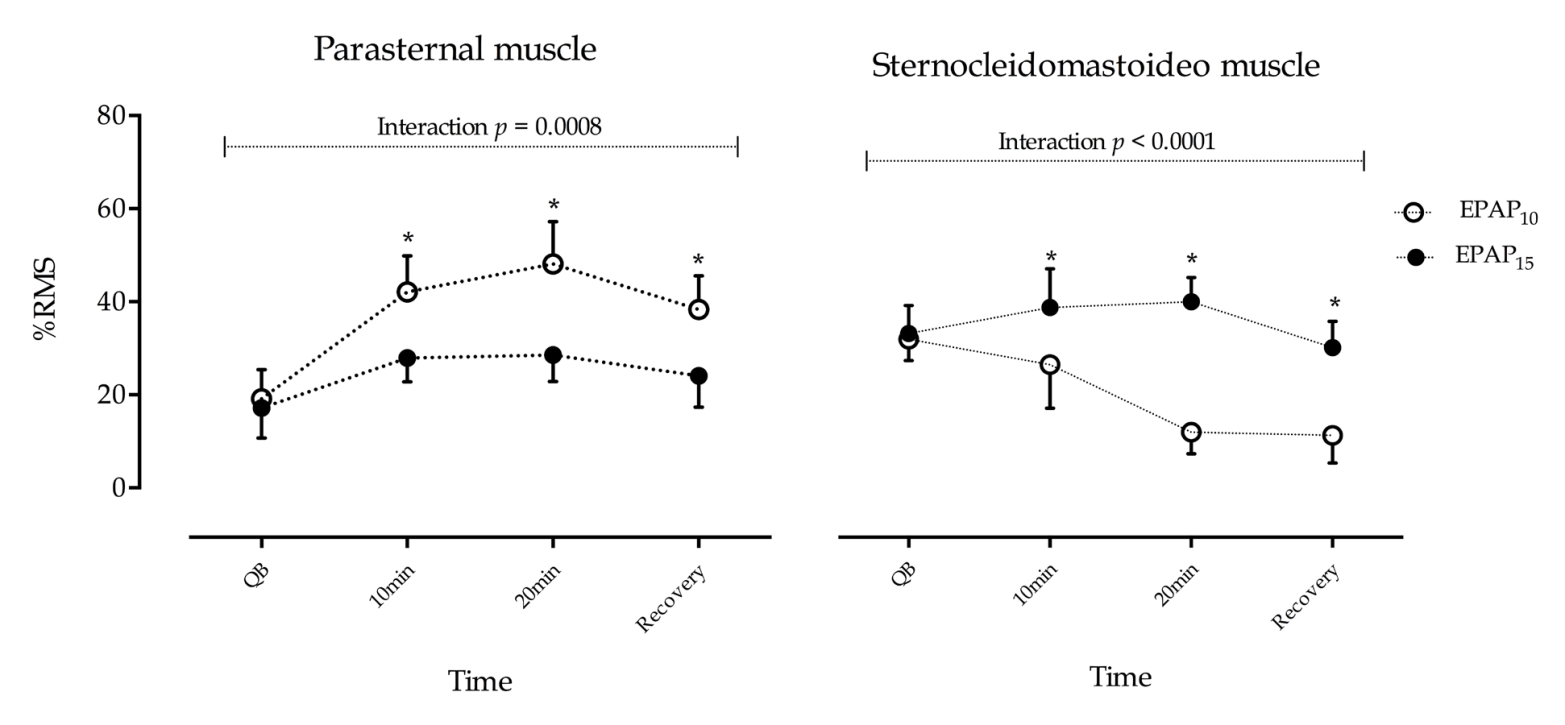
The *sEMG* activity of the parasternal and sternocleidomastoid (SCM) muscles in COPD Patients during the use of EPAP_10_ and EPAP_15_. *p<0.001.

An increase of *sEMG* activity of the parasternal muscle during the use of EPAP was observed in both groups (Figure 2); however, a significant difference between EPAP_10_ and EPAP_15_ was observed because of the influence of intensities and time interaction (*p*=0.008). The *sEMG* activity of the parasternal muscle exhibited a further increase during EPAP_10_. Post-hoc *Bonferroni* analysis showed a significant difference between 10 minutes, 20 minutes, and recovery between EPAP_10_ and EPAP_15_ (*p*<0.001). In relation to baseline, normalized values during EPAP_10_, parasternal muscle activity increased by 23.9% at 10 minutes, 28.9% at 20 minutes, and 19.1% upon recovery compared to increases of 10.7% at 10 minutes, 11.4% at 20 minutes and 6.9% upon recovery during EPAP_15_.

Regarding the *sEMG* activity of the SCM muscle, the pattern that was observed was relatively different from that observed in the parasternal muscle. The electrical activity increased during EPAP_15_ and decreased during EPAP_10_ (Figure 2). A significant difference between EPAP_10_ versus EPAP_15_ was observed because of influence of EPAP levels and time interaction (*p*<0.001). *Bonferroni* post-hoc analysis showed a significant difference between 10 minutes, 20 minutes and recovery between EPAP_10_ and EPAP_15_ (*p*<0.001). In relation to baseline values, during EPAP_15_, the SCM muscle activity increased by 4.8% at 10 minutes and 6.1% at 20 minutes and decreased by –4.0% upon recovery compared to decreases of *–*5.6% at 10 minutes, –20.6% at 20 minutes and –21.3% upon recovery during EPAP_10_.

### Effect of EPAP on ventilation parameters

**Figure 3:**
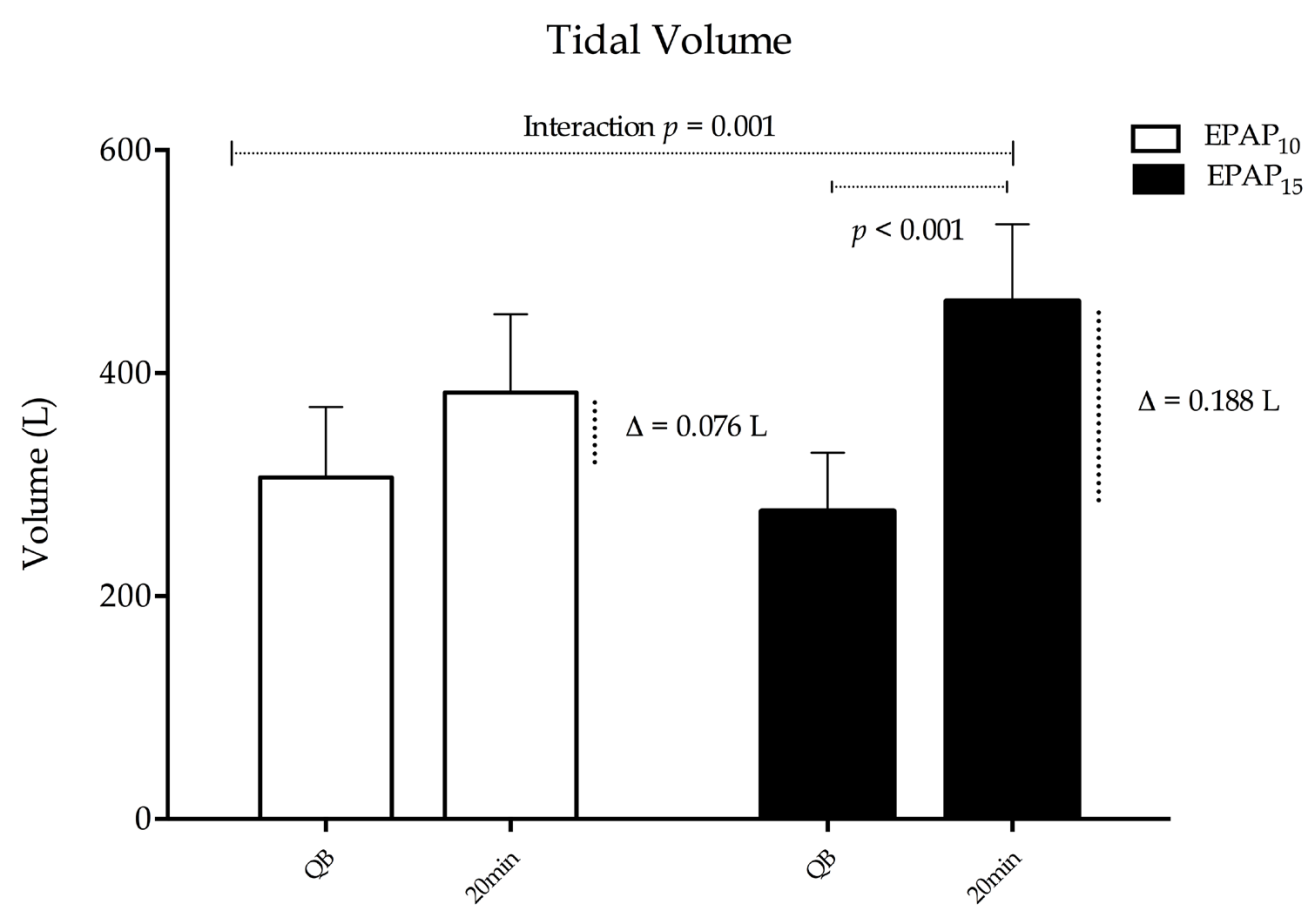
Changes in tidal volume (V_T_) in 2 groups of COPD patients during the use of EPAP_10_ and EPAP_15_.

The application of EPAP induced a significant increase in V_T_ because of interaction between EPAP and time (*p*=0.001, Figure 3). The variation between quiet breathing and 20 minutes of EPAP_10_ and EPAP_15_ were ∆=0.076 L and ∆=0.188 L, respectively. *Bonferroni* post-hoc analysis showed a significant difference between quiet breathing, and 20 minutes of EPAP_15_ use (*p*=0.001).

The V_E_ showed no significant changed in either group (EPAP_10_=5.5±0.4 L to 5.8±0.5 L, versus EPAP_15_=5.2±0.5 L to 5.5±0.6 L, *p*=0.081). In contrast, there was a significant change in SpO_2_ and RR in EPAP_10_ group but just in the RR during EPAP_15_. The decrease in RR was accompanied by a reduction in dyspnea in both groups (Table 2).

**Table 2:** Behavior of physiological variables in 2 groups of COPD patients in the application of EPAP_10_ and EPAP_15_.

Variables	EPAP_10_			EPAP_15_			Between group differences (95% CI) p-value^*^
	*Quite Breathing*	*20 minutes*	*p-value*	*Quite Breathing*	*20 minutes*	*p-value*	
RR (bpm)	18.5±4.1	15.1±2.1	0.013	19.3±3.1	12.0±3.1	<0.001	–3.8 (–7.53 to –0.21) 0.015
SpO_2_ (%)	94.6±2.9	96.0±2.8	0.017	94.7±3.1	96.6±3.4	0.011	–0.5 (–3.23 to 2.22) 0.181
Heart Rate (bpm)	73.1±9.2	80.1±6.4	0.097	76.4±14.1	81.4±12.5	0.043	1.9 (–6.7 to 10.5) 0.649
SBP (mmHg)	118.3±13.1	125.5±20.7	0.413	125.3±18.3	123±15.7	0.541	9.4 (–7.71 to 26.6) 0.261
DBP (mmHg)	77.0±12.1	77.7±12.5	0.759	76.1±6.9	77.1±7.0	0.798	–0.4 (–10 to 9.23) 0.937
Borg’s scale	3.0±0.5	1.5±0.3	0.003	3.1±0.4	0.6±0.2	<0.001	–1.01 (–1.4 to –0.53) 0.028

RR: Respiratory rate; SpO_2_: Saturation peripheral oxygen; SBP: Systolic blood pressure; DBP: Diastolic blood pressure. Data presented as the mean ± standard deviation.

* Comparison between groups. *p*<0.05 was considered to be significant.

## Discussion

The hypothesis that the two different loads of EPAP would proportionally improve the coordination of the respiratory muscles studied and would enhance ventilation was tested. The main findings of this study were partially in agreement with the hypothesis. With an increase in expiratory load, it was expected that the inspiratory muscles would act in coordination, increasing their activity to cope with the expiratory resistance and to improve ventilation. Nevertheless, the results indicated different patterns of *sEMG* activity in the parasternal and SCM muscles during the use of EPAP_10_ and EPAP_15_. While the effect of EPAP_10_ and EPAP_15_ exhibited a similar pattern of increased *sEMG* activity in the parasternal muscles which was more pronounced in EPAP_10_, the effect of EPAP_10_ and EPAP_15_ was different in the SCM muscle. A slight increase in the *sEMG* activity of the SCM muscle was observed during EPAP_10_ while a decrease in the *sEMG* activity was observed during EPAP_15_.

Ventilation at both intensities of EPAP promoted a significant reduction of RR accompanied by an increased tidal volume (V_T_). It is important to state that the increases in ventilatory parameters during EPAP_15_ were greater than those seen during EPAP_10._ However, the different (opposite) pattern of the SCM muscle recruitment during EPAP_15_ use compared to that seen during EPAP_10_ use, appears to be related to the activity employed by the respiratory system to cope with the increase in expiratory load and to promote ventilation. It was not clear why these different responses to EPAP occurred. There were no differences in disease severity among the groups at baseline. Although both absolute and % predicted maximum expiratory volume (MEP) differed between the groups, the differences in SCM activity could not be attributed to the slight decrease in expiratory muscle strength based on maximal static effort. However, it might be possible that parasternal muscle was unable to improve its activity, as observed during EPAP_10_.

As previously described, the EMG activity of the parasternal muscle during application of EPAP_15_ might result from an overload of the inspiratory muscles^27^. This effect was previously described by Simkovitz et al.^28^, who demonstrated that applying positive end expiratory pressure (PEEP) levels greater than 10 cmH_2_O during mechanical ventilation could compromise inspiratory muscle function and increase the operation volumes above levels imposed by the expiratory flow limitation. This would be particularly true if the operation volumes were great enough that the inspiratory muscles function at shorter lengths, a condition that would be less favorable to ventilatory mechanics. However, the EPAP_10_ group showed significant activity in this muscle during therapy and after mask removal. This finding was also observed by O’Donoghue et al.^29^, who examined the effect of extrinsic PEEP (ePEEP) via the application of 1 to 10 cmH_2_O CPAP, on the inspiratory muscle effort of nine patients with severe and stable COPD. These authors found that applying ePEEP in their patients increased the sEMG activity in the parasternal muscle, in addition to reducing the indices of muscle effort, most likely at the expense of a substantial increase in lung volume and expiratory muscle recruitment.

Our study demonstrated that EPAP_10_ reduced SCM muscle activity. This finding was assumed to be the result of the capacity of ePEEP to reduce iPEEP, inspiratory threshold load and respiratory work, even in the absence of a significant increase in lung volume^30^ ^,^ ^31^. The waterfall theory^32^ hypothesized that ePEEP could reduce iPEEP without aggravating hyperinflation only if the latter was caused by expiratory flow limitation, which may have occurred in the present study.

The EPAP_15_ group exhibited greater SCM muscle activity during EPAP therapy. This observation confirmed the results presented by our group in an earlier study^33^, which assessed the effect of EPAP_15_ on the electrical activity of the SCM and scalene muscles in patients with COPD. As in the present study, there was a tendency for greater SCM muscle activity during the application of EPAP, which may have occurred in an attempt to maintain an adequate inspiratory pressure^34^. However, in this study, a significant reduction of SCM electrical activity after the withdrawal of EPAP_15_ was not found, a fact that may have been a result of the insufficient time application of the therapy. Thus, it is also important to consider that the SCM muscle participated in non-respiratory functions, including the maintenance of posture and head movement. De Troyer et al.^35^ found an EMG silent period in the SCM during spontaneous breathing in patients with severe COPD in the supine position, suggesting that respiratory function was the primary function of this muscle.

As expected, V_T_ increased significantly with the application of EPAP_10_ and EPAP_15_. This increase in V_T_ was also observed in a previous study^34^, during the use of 5 and 15 cmH_2_O EPAP applied to healthy subjects. The authors suggested that the higher V_T_ was associated with the level of expiratory pressure that was imposed^34^. This may occur under conditions of higher ventilatory demand, resulting from the greater need for oxygen production or higher carbon dioxide concentration. Conversely^8^, when the effect of 5 cmH_2_O EPAP was assessed in eight men with moderate to severe COPD during exercise; no significant alteration in V_T_ was found, although they suggested that the increase in V_T_ could have been caused by a prolonged expiratory time. These findings are similar to results of our study, where the reduction of RR in both groups was responsible for the increase in V_T_ because the V_M_ did not change significantly.

The findings of the present study could have practical implications and could open a new perspective for EPAP prescription in COPD. There is currently no consensus regarding the adequate pressure levels used as to when the application of elevated PEEP might aggravate ventilatory dysfunction. However, it is important to mention some limitations of this study. The results of V_T_ and RR must be interpreted with caution. It is important to consider that while mechanical respirometers are portable, relatively low cost, precise and accurate devices with worldwide use, they are designed for assessments over short time periods. End expiratory lung volumes (EELVs) were not assessed which is one marker of hyperinflation that could impair ventilation in COPD patients; nonetheless, worsening symptoms, such as dyspnea, were not observed in our study. Additionally, due to the heterogeneity among patients with COPD, new studies with greater subject numbers should be performed. Considering the complexity of respiration, determining the effects of different intensities of expiratory loads on patient comfort and respiratory mechanics through more complex analyses of the chest wall and the symptoms experienced would be appropriate.

In conclusion, in COPD patients, the EPAP_10_ for at least 10 minutes reduces the accessory inspiratory activity of the SCM muscle and increases parasternal muscle activity, which was accompanied by an improvement in ventilation and a reduction in the sensation of dyspnea. The EPAP_15_ improves ventilation more than EPAP_10_, however, the parasternal muscle activity during EPAP_15_ was lower than that observed during EPAP_10_. Patients with COPD adopt different strategies to maintain their ventilation, and the reasons for these differences require further investigation. Based on our results, it is possible to state that EPAP_10_ use, as demonstrated in our study, appears to be more advantageous to patients with COPD.
